# Use of Natamycin for the Development of Polymer Systems with Antifungal Activity for Packaging Applications

**DOI:** 10.3390/polym17050686

**Published:** 2025-03-04

**Authors:** Vincenzo Titone, Manuela Ceraulo, Francesco Lopresti, Giuliana Garofalo, Raimondo Gaglio, Maria Chiara Mistretta, Luigi Botta

**Affiliations:** 1 Department of Engineering, University of Palermo and INSTM Research Unit, V. le delle Scienze, 90128 Palermo, Italy; vincenzo.titone@unipa.it (V.T.); manuela.ceraulo@unipa.it (M.C.); francesco.lopresti01@unipa.it (F.L.); 2 Department of Agricultural, Food and Forest Sciences, University of Palermo, V. le delle Scienze, Bldg. 5, 90128 Palermo, Italy; giuliana.garofalo01@unipa.it (G.G.); raimondo.gaglio@unipa.it (R.G.)

**Keywords:** antifungal activity, biodegradable, circular economy, filamentous fungi, polymers, sustainability

## Abstract

Recently, there has been a rapid growth in the use of biodegradable polymers as alternatives to petroleum-based polymers, particularly in the packaging sector, to reduce environmental pollution. In this scenario, the aim of this work was to study the use of different amounts of Natamycin on two polymer systems: one that is non-biodegradable but widely known in the field of packaging and one that is biodegradable and is emerging as a possible replacement, in order to accelerate progress toward the achievement of the sustainable development goals. Both systems were produced through melt mixing followed by compression moulding. Subsequently, they were fully characterized by rheological, morphological, mechanical, thermal, and wettability analyses. Natamycin release was evaluated in water at 4 °C by UV-Vis measurements. The antifungal activity of both polymeric systems containing Natamycin was assessed in vitro against three strains of undesirable filamentous fungi of food interest. The results show that PCL with 5% Natamycin represents an effective biodegradable alternative to EVA for inhibiting undesirable filamentous fungi. More specifically, both systems at 5% showed comparable inhibition zones of about 30 mm.

## 1. Introduction

Over the years, plastic production has increased dramatically from 15 million tons of plastic in the early 1960s to nearly 420 million tons in 2023 [1]. This unstoppable growth highlights the urgent need for sustainable solutions to address the growing crisis of plastic pollution, which poses a significant threat to the environment [2]. Each year, approximately 22 million tons of plastic products are released into the environment, with around 2.5 million tons converted into microplastics and nanoplastics [3,4].

In response to this crisis, the scientific literature is increasingly pushing the use of biodegradable polymers [5,6,7] as a sustainable solution to replace petroleum-derived polymers. This trend is particularly evident in the packaging industry, where conventional polymers like polyethylene and polypropylene are progressively being replaced by biodegradable counterparts, such as acid polylactide, (PLA) [8], poly(butylene adipate-co-terephthalate) (PBAT) [9], polybutylene succinate (PBS) [10], and others [11]. However, certain commonly used polymers, such as ethylene-vinyl acetate (EVA), still lack effective biodegradable substitutes.

A promising alternative to EVA could be polycaprolactone (PCL), a biodegradable polyester known for its versatility and good mechanical properties. PCL is characterized by a low melting point and high compatibility with various additives, making it suitable for a wide range of applications, including controlled drug release [12,13]. These properties also make PCL a strong candidate for active food packaging [14,15,16], where it can enhance food safety and preservation.

Currently, in the realm of active food packaging, antimicrobial additives such as carvacrol [17], nisin [18], and antifungal compounds are increasingly commonly added to active packaging applications to increase food safety and preservation, as food contamination by fungi threatens human health.

One such compound, Natamycin, also known as pimaricin, a polyene macrolide produced by *Streptomyces natalensis* and *Streptomyces chattanogenesis*, has attracted the attention of the food industry due to its efficacy in controlling fungal growth and mycotoxin production [19,20]. The fungicidal activity of Natamycin is attributed to its high affinity for ergosterol, which is present in the membrane of a eukaryotic cell [21]. Specifically, Natamycin has been described to lead to the formation of polyene–sterol complexes that alter membrane permeability and inhibit fungal growth [22]. Due to its microbial origin, it is regarded as a natural food additive and is classified as Generally Recognized As Safe (GRAS) by the European Food Safety Authority, the World Health Organization, and the Food and Drug Administration [23]. Therefore, the use of Natamycin as an alternative to synthetic chemical preservatives (benzoate, nitrate, nitrite, propionate, sorbate, and sulfites) is permitted to prevent fungal spoilage in foods of both animal and plant origin [24]. Additionally, it is highly preferred over other preservatives because it does not change the color, odor, or taste of foods [25]. Natamycin has demonstrated efficacy at low concentrations against various filamentous fungi that frequently affect food supply chains, such as *Aspergillus* spp., *Cephalosporium* spp., *Cladosporium* spp., *Fusarium* spp., and *Penicillium* spp. [26]. For this reason, it is utilized worldwide in the production and preservation of baked goods, dairy products, fruit and vegetable beverages, and meat products [27]. Moreover, recent studies show that Natamycin is beginning to be incorporated into polymer matrices through casting and surface modification processes [23,28,29,30,31,32] in order to develop active packaging that extends the shelf life of food products, thereby reducing economic losses due to fungal spoilage.

In this regard, the objective of this study is to develop polymeric films loaded with different amounts of Natamycin to evaluate their structure–process–property relationships and to investigate whether the biodegradable matrix can achieve comparable release kinetics and antifungal activity to widely used systems in the packaging industry. This research aims to create a more environmentally friendly packaging solution, contributing to the advancement of sustainable development goals.

## 2. Materials and Methods

### 2.1. Materials

**Figure 1:**
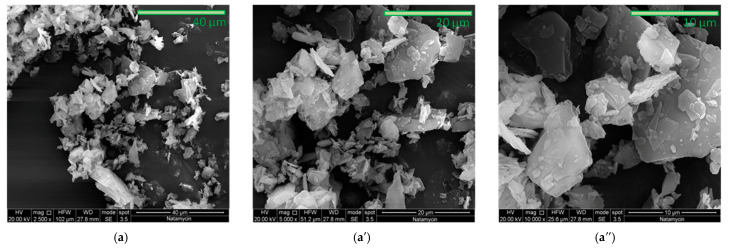
SEM micrographics of the Natamycin at different magnifications: (**a**) 2500×; (**a′**) 5000×; and (**a″**) 10,000×.

The main materials used in this paper are as follows:

- Ethylene-vinyl acetate (EVA) with the trade name Greenflex ML 60 was purchased from Versalis (Versalis, Milan, Italy). It is an ethylene-vinyl acetate copolymer with a melt flow index of 2.5 dg/min, a melting temperature of 74 °C, 28% vinyl acetate, and a density of 0.952 g/cm^3^.
- Polycaprolactone (PCL) was purchased from Sigma Aldrich (Sigma Aldrich, St. Louis, MO, USA) with a molecular weight of 80 kDa and a density of 1.145 g/cm^3^.
- Natamycin (NAT) also known as Pimaricin was supplied by Handary (Handary, Brussels, Belgium). Figure 1 shows an observation by SEM images at different magnifications of Natamycin used in this work.

Figure 1 is presented at different magnifications to highlight the morphology of Natamycin, which shows small dimensions with irregular and slightly prismatic morphology. The filler is shown to better illustrate its characteristics in the context of the work.

### 2.2. Development of Antifungal Films for Packaging Applications

EVA and PCL systems at different Natamycin contents (0, 1, 3, and 5%) were prepared using a melt mixer Brabender mod. PLE330 (Brabender, Duisburg, Germany) operating at 100 °C at 60 rpm for 5 min in accordance with what is reported in the literature on Natamycin [33]. Before use, PCL was dried at 40 °C overnight, while EVA did not undergo any treatment.

All specimens for the subsequent characterizations were obtained using a Carver laboratory hydraulic press (Carver, Wabash, IN, USA) at a temperature of 100 °C and a moulding pressure of 300 psi for about 3 min. The cooling time was 10 min. The films obtained had an average thickness of 200 μm.

**Figure 2:**
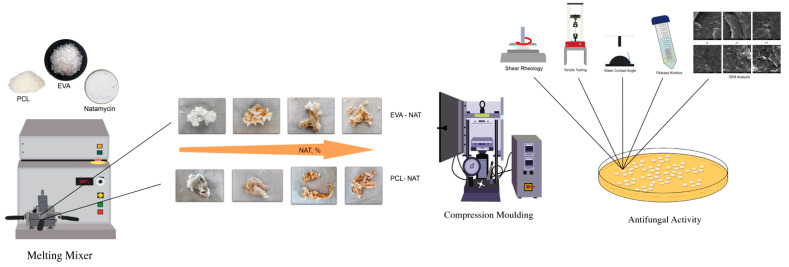
A brief illustration of the process used in this paper.

A brief illustration of the process used is shown in Figure 2.

### 2.3. Characterization

#### 2.3.1. Rheological Characterization

Rheological characterization was performed using an Ares G2 (TA Instrumental, New Castle, DE, USA) with parallel plate geometry. Tests were performed on disk-shaped samples with a diameter of 25 mm. The temperature was set at 100 °C with an angular frequency range of 100 to 0.1 rad/s. The strain used was 5%.

#### 2.3.2. Morphological Characterization

Morphological analysis was performed using an FEI Quanta 200 (FEI Co., Hillsboro, OR, USA) scanning electron microscope (SEM). All specimens were brittle-broken by immersing them in liquid nitrogen for approximately 25/30 min as reported elsewhere [34]. Before analysis, all specimens were gold-spattered to make them conductive.

#### 2.3.3. Mechanical Characterization

Tensile tests were conducted at room temperature (25 ± 3 °C) using an Instron universal testing machine (Instron, mod. 3365, High Wycombe, UK) according to ASTM D638 [35]. The specimens were rectangular in shape with the following sizes: 90 × 10 × 0.20 mm. The crosshead speed was set at 1 mm/minute up to 3% of elongation and 100 mm/min until failure. Elastic modulus, E, tensile strength, TS, and elongation at break, EB, were the outcomes of the tests. The mean value of the mechanical tests with the corresponding standard deviation is the result of eight measurements.

#### 2.3.4. Differential Scanning Calorimetry Analysis

A differential scanning calorimeter (Setaram, model DSC131, Lyon, France) was used to assess the thermal characterization of the materials. The amount of sample placed in the DSC aluminum pans was about 7 ± 2 mg, while the heating rate was from 10 °C/min up to 120 °C. The following Equation (1) was adopted for evaluating the degree of crystallinity, *Xc*

(1) $$Xc,\;\%=\frac{{∆H}_{m}}{{∆H}_{polymer}^{0}\times X_{polymer}}$$

where Δ*H_m_* is the melting enthalpy of the samples; *X_polymer_* is the weight fraction of polymer in the composite; and Δ*H^0^_polymer_* is the 100% crystalline polymer melting enthalpy. For EVA, it is 281 j/g [36], while for PCL, it is 136 j/g [37].

#### 2.3.5. Surface Analysis

The surface wettability was evaluated at room temperature (25 ± 3 °C) using an FTA 1000 (First Ten Ångstroms, Cambridge, UK). The method used was the sessile drop method [38]. The time of measurement was 20 s. In all cases, based on each image, the software calculated the contact angle. The tests were performed in triplicate.

#### 2.3.6. Natamycin Release Kinetic

The Natamycin (Nat) release curve was assessed by immersing a pre-weighed sample (20 × 10 mm square, weight ~100 mg) in 10 mL of distilled water at 4 °C. The absorbance of Nat (maximum absorbance peaks were detected at 320 nm) was measured at different time points, and the concentration of the compound was evaluated using a calibration line. The calibration line was obtained by preparing and analyzing different solutions containing 1 to 50 mg/L of Nat by UV-Vis (Specord 250 plus, Analytik Jena, Bucharest, Romania) and correlating the absorbance peak intensity with the concentration of Nat (mg/L).

#### 2.3.7. Determination of Antifungal Activity

The antifungal activity of polymeric systems containing Natamycin was performed against three strains of filamentous fungi belonging to the species *Alternaria tenuissima* 1.8 (Ac. No. KM265454), *Aspergillus niger ML168B* (Ac. No. KC692215), and *Penicillium olsonii* 1.17 (Ac. No. KM265447), following the approach described by [39] with some modifications. Fungal colonies were propagated on Potato Dextrose Agar (PDA) (Condalab, Madrid, Spain) and incubated at 25 °C until they completely covered the plate. After growth, 5 mL of peptone solution (1 g/L) was added to the colonies, which were then scraped with a sterile loop (Biosigma S.r.l., Cona, Italy) to facilitate the conidial dispersion. The resulting fungal suspensions were filtered through cell strainers (Merck KGaA, Darmstadt, Germany) to separate the conidia from the mycelium. One hundred microliters of suspensions at the final concentration of 10^6^ conidia per milliliter of each strain were spread and plated on PDA. Then, discs of each polymeric system, which were 0.6 cm in diameter, were placed on the surface of the agar plates. Polymeric systems without Natamycin were used as a negative control, while 0.6-cm diameter sterile paper discs (Whatman^®^ qualitative filter paper, Grade 1, Maidstone, UK) were soaked with 5 μL of cicloheximide (10 mg/mL) (Sigma-Aldrich, St. Louis, MO, USA) and used as a positive control. Antifungal activity was assessed after incubation for 7 d at 25 °C by measuring the diameter of the inhibition zone around the discs with a digital caliper (Stainless Hardened, Farnell, Poland). This analysis was carried out in triplicate.

## 3. Results and Discussion

### 3.1. Physical and Structural Characterization

**Figure 3:**
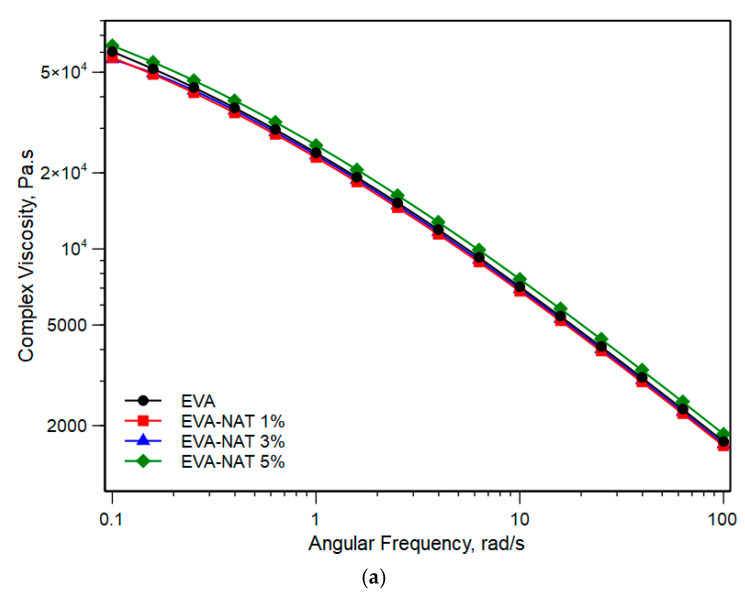

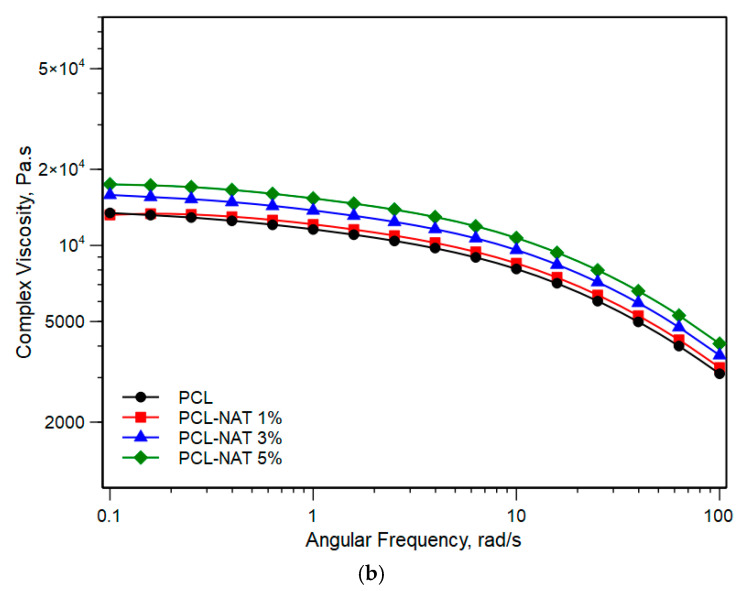
(**a**) Complex viscosity versus angular frequency of EVA and EVA/Natamycin systems. (**b**) Complex viscosity versus angular frequency of PCL and PCL/Natamycin systems.

In order to obtain information on its processability, rheological tests were performed to evaluate the influence of Natamycin on the viscoelastic behavior of systems. Figure 3a,b displays the complex viscosity curves of EVA and PCL with their Natamycin systems, respectively.

From Figure 3a,b, it is evident that the two polymers show a different rheological behavior. Specifically, EVA does not exhibit a typical Newtonian plateau at a low frequency but does exhibit a non-Newtonian behavior that becomes more pronounced at a high frequency. In contrast, PCL exhibits Newtonian behavior at a low frequency and non-Newtonian behavior at high frequencies. However, the η0 at 0.1 rad/s of the EVA shows higher values than PCL. The addition of Natamycin causes both EVA and PCL to slightly increase their viscosity as the amount of Natamycin increases. This result has already been observed in other similar systems [40,41] and could be attributed to the micro-filler action of this solid agent that causes an increase in the melt viscosity.

**Figure 4:**
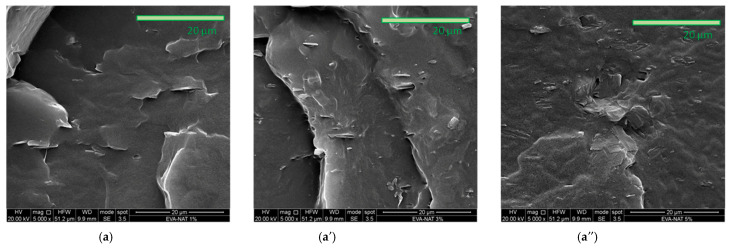

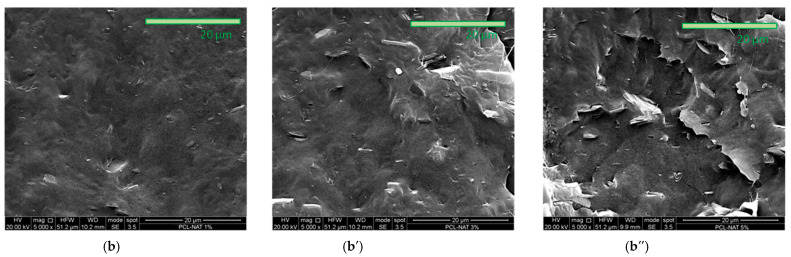
SEM micrographs of all systems investigated: (**a**) EVA-NAT 1%, (**a′**) EVA-NAT 3%, (**a″**) EVA-NAT 5%, (**b**) PCL-NAT 1%, (**b′**) PCL-NAT 3%, (**b″**) PCL-NAT 5%.

Figure 4a,b shows the fracture surface of EVA-NAT (a, a′ and a″) and PCL-NAT films (b, b′ and b″) with 1, 3, and 5% of Natamycin.

The SEM images of the film show a change in morphology with increasing Natamycin content in both systems. At 1%, the surfaces appear smooth and free of aggregates and cracks, and the presence of Natamycin is difficult to distinguish, suggesting an exceedingly fine distribution. At 3%, an even distribution of Natamycin and good interfacial adhesion are observed, evidenced by the absence of voids or defects. This result is consistent with similar studies in the literature [42,43], which also report similar behavior at similar additive concentrations. However, at higher concentrations, the surfaces exhibit slight irregularities, likely due to the formation of small aggregates and irregularly shaped structures, which can be attributed to the nature of Natamycin, as also observed in the Natamycin micrograph images reported in Figure 1. However, overall SEM micrographics indicate that the Natamycin content does not significantly alter the morphology, demonstrating good structural homogeneity.

**Figure 5:**
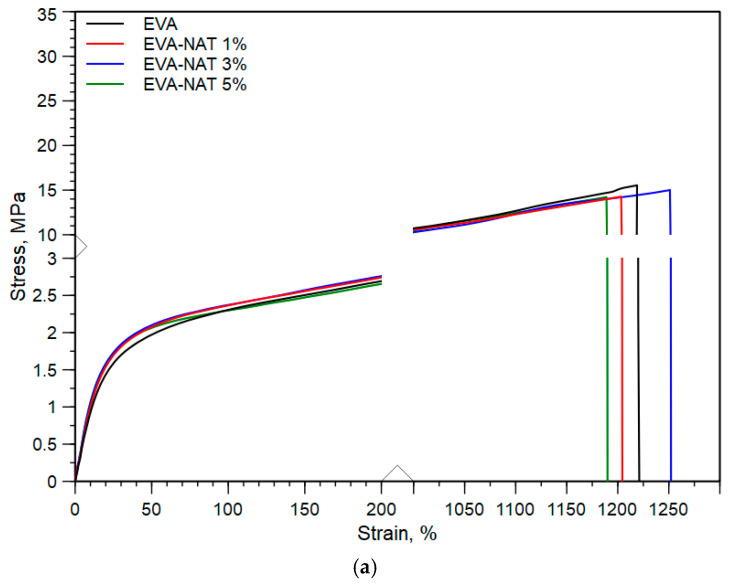

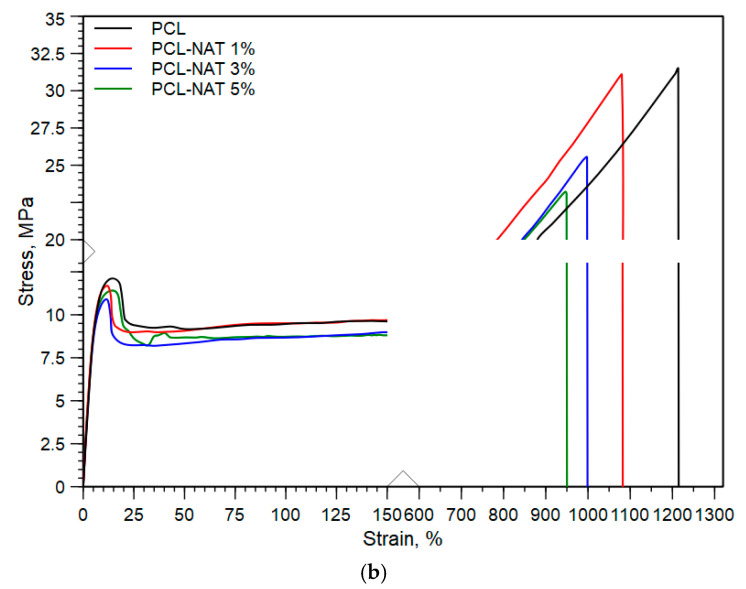
(**a**) Typical stress–strain curves of EVA and EVA/Natamycin systems. (**b**) Typical stress–strain curves of PCL and PCL/Natamycin systems.

Tensile tests are an important parameter in assessing the quality of the packaging material. To this end, Figure 5a,b displays the typical stress–strain curves of EVA and PCL with their Natamycin systems, respectively, while the average values of elastic modulus, E, tensile strength, TS, and elongation at break, EB, with their respective standard deviation, are summarized in Table 1.

As can be seen from the stress–strain curve, EVA is a flexible and ductile material at room temperature (25 ± 3 °C), with an elastic modulus of 9.5 MPa, tensile strength of 14.3 MPa, and elongation at break of 1172% (see Table 1). On the other hand, PCL is less flexible than EVA, while still showing good stretchability. Specifically, PCL at room temperature (25 ± 3 °C) showed an elastic modulus of 216 MPa, tensile strength of 31.5 MPa, and elongation at break of 1214%. The addition of Natamycin, as visible from Table 1, in both EVA and PCL showed a slight increase in elastic modulus with increasing content, without a drastic reduction both in tensile strength and, in particular, in elongation at break. In fact, the maximum reduction in elongation at break at higher Natamycin content was observed for PCL-NAT 5% and was approximately 20%. This behavior could be explained by good interfacial adhesion of the Natamycin with the matrices, as observed from the previous SEM image, resulting in a stiffer system with good stretchability.

Table 2 summarizes the melting temperatures, enthalpies of melting, and crystallinity values obtained from the first scan DSC thermograms for all systems analyzed.

As is clearly visible in Table 2, in both systems, the melting temperature remained almost constant as Natamycin increased. In parallel, instead, it is possible to observe a slight increase in crystallinity as the content of Natamycin content. These results, in keeping with the results of tensile tests and in agreement with other similar systems reported in the literature [29,44], can be attributed to the fact that Natamycin acts slightly as a nucleating agent for both EVA and PCL.

In addition to mechanical properties, when considering these materials for packaging applications, another important test is the water contact angle. Accordingly, the average values and respective standard deviations of the water contact angle of PCL and EVA and their system with Natamycin are summarized in Table 3.

It can be seen in Table 3 that the two polymers exhibit slightly different wettability. In particular, EVA exhibits lower contact angle values than PCL, consequently showing slightly higher wettability. In detail, the contact angle value of EVA is 72 ± 2, while that of PCL is 67 ± 2. In both cases, the addition of Natamycin increased with increasing content of contact angle values in all systems analyzed, suggesting a decrease in wettability. This result, in agreement with the literature results [45,46], could be attributed to the irregularity and roughness of the surface of the films caused by the addition of Natamycin.

### 3.2. Natamycin Release Kinetics

**Figure 6:**
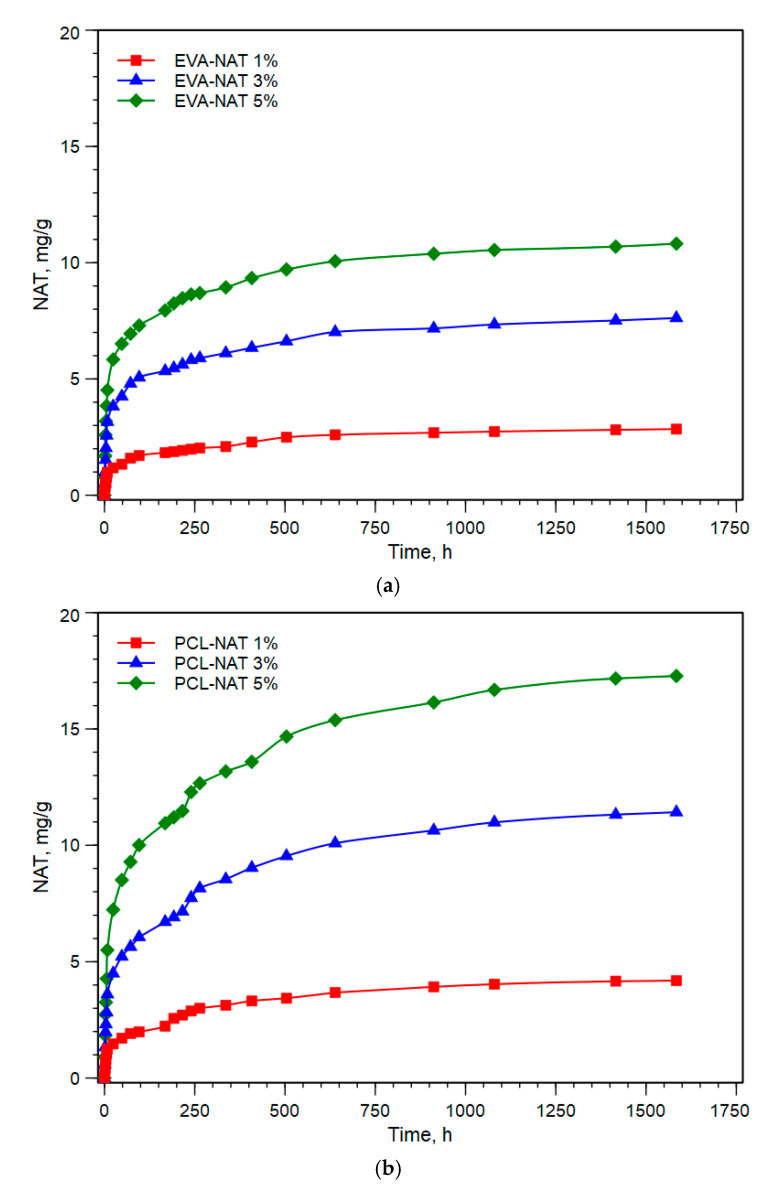
(**a**) Cumulative Natamycin release of EVA-NAT. (**b**) Cumulative Natamycin release of PCL-NAT.

In order to evaluate the release kinetics of Natamycin, the release tests in water were conducted. Figure 6a,b shows the cumulative release of Natamycin of EVA-NAT and PCL-NAT, respectively.

As visible from Figure 5a,b, both EVA and PCL showed a two-phase release characterization: a burst phase in the first hour of release and a second phase characterized by a slower rate of release, reaching an equilibrium at approximately 1000 h. However, an important aspect to note is that the amount of Natamycin release differs between the two polymers. In fact, although the amount of Natamycin released increased proportionally to the increase in the amount incorporated, PCL released more Natamycin than EVA over time. In detail, the increase in % of Natamycin release at 1, 3, and 5% saturation (approximately 1500 h) is 47, 49, and 59%, respectively. This phenomenon could be attributed to the different chemical and physical properties of the two polymers. However, despite the fact that Natamycin remains trapped in the EVA matrix longer and achieves a longer-lasting antifungal effect, the results of PCL show that this polymer can be a valid replacement for EVA, widely known in the packaging industry, with advantages in terms of both antifungal releases and therefore activity antifungal and sustainability.

### 3.3. Antifungal Activity

**Figure 7:**
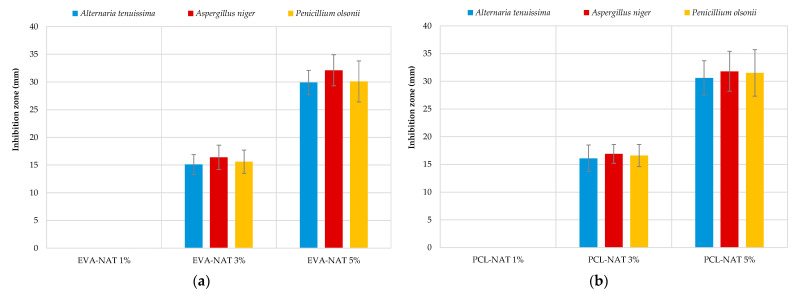
Antifungal activity of EVA and PCL systems with different Natamycin concentrations. (**a**) Ethylene-vinyl acetate (EVA) systems; (**b**) Polycaprolactone (PCL) systems.

The strains used to evaluate the antifungal activity of EVA and PCL systems with different Natamycin concentrations belonged to the species *Alt. tenuissima*, *Asp. niger*, and *P. olsonii*. These filamentous fungi were selected because they are known to cause food spoilage [47]. As shown in Figure 7a,b, the antifungal activity of both polymeric systems increased significantly with the increasing percentage of Natamycin added. In particular, the tests showed inhibition zones around the discs for all three microorganisms, ranging from approximately 15.0 mm for EVA-NAT 3% and PCL-NAT 3% to 30.0 mm for EVA-NAT 5% and PCL-NAT 5%. This confirms Natamycin’s ability to inhibit fungal growth by targeting specific components of fungal cell membranes (binding to ergosterol) [48].

Due to the lack of studies reporting the antifungal activity of EVA and PCL material containing Natamycin, a direct comparison with literature data is not possible. However, similar trends have been reported by several authors [49,50,51] for other typologies of Natamycin-activated packaging systems. Our results suggest that PCL containing Natamycin is an effective biodegradable alternative to EVA, capable of inhibiting filamentous fungi that cause food spoilage.

## 4. Conclusions

In this paper, different amounts of Natamycin were incorporated through melt-mixing into ethylene-vinyl acetate (EVA) and Polycaprolactone (PCL). The use of different amounts of Natamycin on physical and structural properties as well as Natamycin release and antifungal activity against *Alternaria tenuissima*, *Aspergillus niger*, and *Penicillium olsonii* was studied. The results show that Natamycin causes a slight increase in viscosity with increasing amounts in both systems, likely attributable to the micro-filler action. On the other hand, morphological analysis revealed good matrix/Natamycin interfacial adhesion, as evidenced by the absence of voids and surface defects. In terms of mechanical properties, the results indicate that Natamycin allows us to obtain stiffer systems with good stretchability (maximum reduction observed was 20%). Moreover, a slight nucleating action and a slight increase of approximately 10% in wettability were observed for both systems. From a kinetic release point of view, Natamycin release increased proportionally to the increase in the amount incorporated, with PCL releasing more Natamycin than EVA over time. In conclusion, the overall results show that PCL can be considered a good replacement for EVA, with super imposable antifungal activity.

## Figures and Tables

**Table 1:** Average values of elastic modulus, E, tensile strength, TS, and elongation at break, EB, of PCL, EVA, and their systems with Natamycin.

Sample Code	E, MPa	TS, MPa	EB, %
EVA	9.5 ± 0.6	14.3 ± 0.9	1172 ± 69
EVA—NAT 1%	9.8 ± 0.7	14.6 ± 1.0	1170 ± 60
EVA—NAT 3%	11.1 ± 0.8	14.9 ± 1.4	1219 ± 71
EVA—NAT 5%	11.9 ± 0.9	14.2 ± 1.2	1124 ± 102
PCL	216 ± 15	31.5 ± 2.2	1214 ± 95
PCL—NAT 1%	226 ± 7.2	31.1 ± 2.3	1080 ± 60
PCL—NAT 3%	234 ± 19	26.0 ± 2.4	1046 ± 103
PCL—NAT 5%	256 ± 20	21.1 ± 2.1	960 ± 27

**Table 2:** Melting temperature, Tm, melting enthalpy, ΔH, and crystallinity, Xc%, values of all systems investigated, obtained from the first scan DSC thermograms.

Sample Code	Tm, °C	ΔH, J/g	Xc, %
EVA	75.2 ± 0.1	12.7 ± 0.9	4.50 ± 0.32
EVA—NAT 1%	75.3 ± 0.2	12.6 ± 1.0	4.52 ± 0.35
EVA—NAT 3%	75.3 ± 0.2	12.4 ± 1.4	4.54 ± 0.51
EVA—NAT 5%	75.5 ± 0.3	12.2 ± 1.2	4.57 ± 0.44
PCL	58.7 ± 0.1	67.8 ± 2.1	49.8 ± 1.61
PCL—NAT 1%	58.8 ± 0.2	67.8 ± 2.2	50.3 ± 1.63
PCL—NAT 3%	58.6 ± 0.1	66.7 ± 2.5	51.9 ± 1.89
PCL—NAT 5%	58.5 ± 0.2	68.7 ± 2.7	53.1 ± 2.08

**Table 3:** Average values of water contact angle, WCA, of PCL, EVA, and their systems with Natamycin.

Sample Code	WCA, %
EVA	72 ± 2
EVA—NAT 1%	74 ± 1
EVA—NAT 3%	75 ± 2
EVA—NAT 5%	77 ± 2
PCL	67 ± 2
PCL—NAT 1%	75 ± 1
PCL—NAT 3%	76 ± 2
PCL—NAT 5%	77 ± 2

## Data Availability

Data are contained within the article.
